# Reinforcement Learning-Based Joint User Pairing and Power Allocation in MIMO-NOMA Systems

**DOI:** 10.3390/s20247094

**Published:** 2020-12-11

**Authors:** Jaehee Lee, Jaewoo So

**Affiliations:** Department of Electronic Engineering, Sogang University, Seoul 04107, Korea; jaeheelee@sogang.ac.kr

**Keywords:** non-orthogonal multiple access, multiple-input multiple-output, user pairing, power allocation, reinforcement learning

## Abstract

In this paper, we consider a multiple-input multiple-output (MIMO)—non-orthogonal multiple access (NOMA) system with reinforcement learning (RL). NOMA, which is a technique for increasing the spectrum efficiency, has been extensively studied in fifth-generation (5G) wireless communication systems. The application of MIMO to NOMA can result in an even higher spectral efficiency. Moreover, user pairing and power allocation problem are important techniques in NOMA. However, NOMA has a fundamental limitation of the high computational complexity due to rapidly changing radio channels. This limitation makes it difficult to utilize the characteristics of the channel and allocate radio resources efficiently. To reduce the computational complexity, we propose an RL-based joint user pairing and power allocation scheme. By applying Q-learning, we are able to perform user pairing and power allocation simultaneously, which reduces the computational complexity. The simulation results show that the proposed scheme achieves a sum rate similar to that achieved with the exhaustive search (ES).

## 1. Introduction

5G mobile communication is further increasing the number of users using the wireless Internet. Moreover, autonomous vehicles connected to 5G are also increasing. Hence, the importance of spectrum efficiency has been significantly increasing, and non-orthogonal multiple access (NOMA) is one of most important research areas [1]. NOMA techniques can be categorized into two main classes: power-domain and code-domain NOMA. Code-domain NOMA is a technique for multiplexing users based on “codeword.” The concept of code-domain NOMA was inspired by the classic code division multiple access (CDMA) system [2]. Code-domain NOMA allows multiple users to share the same time-frequency resources but adopts unique user-specific spreading sequences. The spreading sequences are restricted to sparse sequences or non-orthogonal low cross-correlation sequences in code-domain NOMA. Sparse code multiple access (SCMA) is one of the most important techniques in recent code-domain NOMA. In particular, studies to improve spectral efficiency by using low density parity check (LDPC) codes is actively being conducted [3,4]. Power-domain NOMA is another technique that allows multiple user equipment (UEs) to access the same time/frequency resource, where the signals from the UEs are multiplexed through different power allocation coefficients [5]. The transmit power at the base station (BS) is divided up between the UEs. UEs with poor channel conditions receive more transmit power, whereas UEs with better channel conditions receive less transmit power. On the receiver side, successive interference cancellation (SIC) is used to recover each communication. The SIC successively decodes and subtracts the received signal until it reaches its desired signal [5]. SIC and power allocation are important techniques in power-domain NOMA systems. In this paper, we study the user pairing and power allocation for power-domain NOMA systems.

Multiple-input multiple-output (MIMO) is another technique for enhancing the spectrum efficiency. The application of MIMO to NOMA can result in an even higher spectral efficiency. We consider user pairing and power allocation in MIMO-NOMA systems. Many researchers have already investigated user pairing or power allocation in MIMO-NOMA systems [6,7,8,9,10,11,12]. In [6], a joint user pairing and power allocation scheme in virtual MIMO systems was proposed. First, power allocation was performed with known paired user groups, and power allocation was solved with a multi-level water-filling method. In the next step, joint user pairing and power allocation were conducted with an iterative algorithm based on the analysis in the first step. In [7], the authors proposed user pairing and scheduling algorithms for massive MIMO–NOMA systems to maximize the sum rate by mitigating inter-pair interference. In [8], an optimal NOMA power allocation scheme for improving the spectrum efficiency of coexisting multi-user (MU)-MIMO and orthogonal multiple access (OMA) device-to-device (D2D) networks was proposed. In [9], a 2-user downlink MIMO–NOMA power allocation scheme was proposed. The non-convex MIMO–NOMA power allocation problem was formulated with optimal and suboptimal solutions. Furthermore, an optimal power allocation scheme for maximizing fairness was proposed in [10]; all UEs have the same data rate based on the max–min rate criteria power allocation scheme. In [11], user pairing was combined with power allocation in downlink NOMA systems. The UEs were sorted according to the channel gain, and then the optimal power allocation was applied to enhance the spectrum efficiency. In [12], the authors proposed a user pairing and power allocation scheme in a NOMA system, where the number of users is limited to two. In [8,9,10,11,12], power allocation schemes are proposed for NOMA systems. The conventional schemes formulated the power allocation problem based on convex optimization and tried to find the power by mathematically solving the convex problem. However, we apply the RL to determine the power of the UEs in each pair in the MIMO-NOMA system. Moreover, while the conventional schemes required a high computational complexity to determine the user pairing and power allocation in a MIMO-NOMA system, we find the user pairing and power allocation at the same time with low computational complexity.

Many researchers have applied deep learning (DL) in wireless communication [13,14]; the method includes supervised, unsupervised, and reinforcement learning (RL). Supervised learning requires many datasets for training, which may make it difficult to apply to real-time wireless communication environments. In unsupervised learning, data are classified, or statistical distributions are estimated, and user pairing and power allocation are difficult steps. Another learning method is the Q-learning (one of the RL techniques) which is a widely used model-free RL technique. The Q-learning can solve a user pairing and power allocation problem through action. The channel state information (CSI) between the UE and BS changes continuously at every time slot owing to the movement of UEs or shadowing between buildings. Therefore, Q-learning, which determines the optimal reward by applying CSI without a dataset, may be more suitable for wireless communications than other supervised learning techniques that require many datasets.

Some researchers have applied DL to NOMA systems [15,16,17,18,19,20,21,22,23,24,25]. In [15], a DL-aided sparse code multiple access (SCMA) was proposed in which the mapping of data to the resource and the decoding of received signals is conducted with a deep neural network (DNN). In [16], the authors proposed a deep RL-based power allocation with a dual DNN to overcome the noisiness/randomness problem in training data. Moreover, in [17], the NOMA channel was estimated by applying long short-term memory (LSTM), which is used to learn the CSI of the NOMA system through offline and online training. The authors in [18,19] proposed a fast RL method with a (τ,ϵ)-greedy based deep Q network (DQN) in jamming environments. Furthermore, user pairing was achieved in [20] by applying multi-agent RL to a multi-carrier NOMA system. In [21], the authors proposed a DQN-based joint power allocation and channel assignment for NOMA systems. They derived a closed-form solution for power allocation, where they proposed an attention based DQN for the channel assignment problem. In [22], the dynamic channel access problem was formulated as a partially observable Markov decision process (POMDP), and DQN was applied to find the access policy via online learning. In [23], the authors proposed a multi-agent DNN approach to predict spectrum occupation of unknown neighbouring networks in slotted wireless networks, where they trained the DNN in an online way, using both RL and supervised learning. The authors in [24] proposed a DQN-based power allocation for a multi-cell network to maximize the total network throughput. In [25], a joint precoding and SIC decoding scheme for MIMO–NOMA system was presented in the imperfect SIC decoding environment.

The key challenges in MIMO-NOMA are beamforming, optimization, power allocation, user pairing, and SIC ordering. These challenges have been studied jointly or partially, under specific performance metrics. MIMO-NOMA is a technology that can enhance spectral efficiency in 5G, but it has a fundamental limitation of high computational complexity. This paper aims to increase the sum rate and reduce the computational complexity by using the RL-based joint power allocation and user pairing in MIMO-NOMA systems. The contributions of this paper are as follows: First, we propose an RL-based joint user pairing and power allocation scheme for MIMO-NOMA systems. The previous studies independently investigated user pairing and power allocation problems; or they researched user pairing and power allocation problems via mathematical approaches such as convex optimization in a simplified system with a few users. To the best of the authors’ knowledge, this study is the first attempt in which RL is applied to perform user pairing and power allocation jointly under a practical system with multiple users. Second, the proposed RL-based scheme reduces the computational complexity. In the conventional schemes, the user pairing is performed after the BS has received information about the location and CSI from UEs, and then the power is allocated to UEs in each pair. In this paper, the user pairing and power allocation are simultaneously performed through RL when a BS receives the location and CSI from UEs. Exhaustive search (ES) is a scheme to find the maximum sum rate, but its computational complexity is extremely high because it finds all pairs that can be user paired, calculates all the coefficients that can be power allocation, and then finds the sum rate. The proposed RL scheme reduces the computational complexity because the sum rate is calculated with one action selection. Third, the proposed RL-based scheme shows that the sum rate is superior to those of OMA and other comparable schemes. The proposed scheme at the beginning of the simulation shows that the sum rate is low because the BS randomly selects the action, but as the time slot increases, the learning proceeds and it approximately converges to the sum rate of the ES. Moreover, it was shown that the proposed scheme is more efficient than the ES or phased RL schemes in terms of the time and computational complexity.

The remainder of this paper is organized as follows: Section 2 describes the system model, and Section 3 presents the proposed RL-based joint user pairing and power allocation in MIMO-NOMA systems. The numerical results are presented in Section 4 and Section 5 concludes this paper.

For the sake of clarity, the main symbols and their descriptions used in this paper are summarized in Table 1.

*Notations:* Vectors are presented by boldface small letters, while matrices are represented by boldface capital letters; $I_{N}$ is the Identity matrix and $\hat{h}$ the quantized value of *h*.

## 2. System Model

### 2.1. System Description

**Figure 1:**
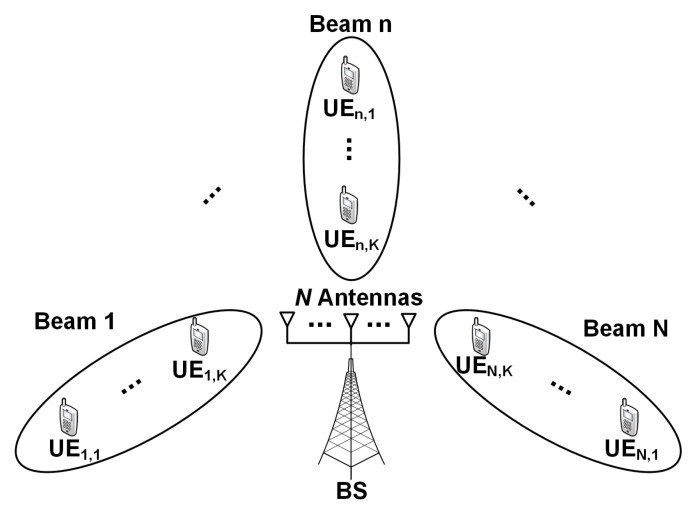
System model.

In this paper, we consider a downlink MIMO–NOMA in a macro cell with 500 m radius, as shown in (Figure 1). The BS has $P_{BS}$ transmit power, and it allocates the same power to the *N* antennas. Thus, BS transmits a superimposed signal, considering the characteristics of NOMA. To create a MIMO–NOMA applicable scenario, all *M* UEs are randomly distributed in a cell. The transmitted power at each beam can be expressed as $P_{n}=\frac{P_{BS}}{N}$. We assume that the channel gain is ordered as follows:

(1) $$|h_{n,i}{|}^{2}\leq {\left|h_{n,j}\right|}^{2},\;\;\;\mathrm{for}\;i\leq j.$$

In NOMA, the UE close to the BS can cancel the interference signal by using SIC, where the interference signal may be the signal sent to the UE with poor channel conditions. Here, the SIC is assumed to be operated with little or no errors. In addition, the BS is responsible for pairing UEs and then it determines the transmit power of each UE. Each UE suffers from Rayleigh fading and additive white Gaussian noise (AWGN) with zero mean and variance $\sigma_{n,k}$. The superimposed signal transmitted by the BS is as follows:

(2) $$\begin{array}{c} x_{n}=\sum_{k=1}^{K}\sqrt{\alpha_{n,k}P_{n}}s_{n,k}, \end{array}$$

where $s_{n,k}$, $\alpha_{n,k}$, $P_{n}$ denote the signal transmitted by the BS, the power allocation coefficient, and the transmit power of each beam, respectively. The signal received at the UE${}_{n,k}$ is as follows:

(3) $$y_{n,k}=h_{n,k}\sum_{n=1}^{N}w_{n}x_{n}+n_{n,k},$$

where $h_{n,k}$ is the Rayleigh fading channel vector from the BS to the UE${}_{n,k}$, $w_{n}$ is the precoding vector for each beam in the precoding matrix $W=\left[w_{1},w_{2},\cdots ,w_{n}\right],w_{n}\in C^{1\times \;N}$, and $n_{n,k}$ is the AWGN; $h_{n,k}$ can be expressed as follows:

(4) $$\begin{array}{cc} h_{n,k} & =h_{n,k}\sqrt{d_{n,k}^{-\eta }}. \end{array}$$

Moreover, the distance between the BS and UE${}_{n,k}$ is denoted as $d_{n,k}$, the path loss exponent is η, and $h_{n,k}$ represents the RL’s state. Equation (3) can be rewritten as follows:

(5) $$\begin{array}{cc} y_{n,k}= & \;h_{n,k}\sqrt{P_{n}\alpha_{n,k}}s_{n,k}+\underset{\mathrm{intra}-\mathrm{beam}\;\mathrm{interference}}{\underbrace{h_{n,k}w_{n}\sum_{k^{\prime }=k+1}^{K}\sqrt{P_{n}\alpha_{n,k^{\prime }}}s_{n,k^{\prime }}}}\\ \; & +\underset{\mathrm{inter}-\mathrm{beam}\;\mathrm{interference}}{\underbrace{h_{n,k}\sum_{n^{\prime }=1,n\neq n}^{N}w_{n^{\prime }}x_{n^{\prime }}}}+n_{n,k}. \end{array}$$

After SIC, Equation (5) can be rewritten as follows:

(6) $$\begin{array}{cc} y_{n,k}= & \left\{\begin{array}{cc} \; & h_{n,k}\sqrt{P_{n}\alpha_{n,k}}s_{n,k}+h_{n,k}\sum_{n^{\prime }=1,n\neq n}^{N}w_{n^{\prime }}x_{n^{\prime }}+n_{n,k},\;\;\mathrm{if}\;\;k=K,\;\\ \; & h_{n,k}\sqrt{P_{n}\alpha_{n,k}}s_{n,k}+h_{n,k}w_{n}\sum_{k^{\prime }=k+1}^{K}\sqrt{P_{n}\alpha_{n,k^{\prime }}}s_{n,k^{\prime }} \end{array}\right.\\ \; & \;\;\;+h_{n,k}\sum_{n^{\prime }=1,n\neq n}^{N}w_{n^{\prime }}x_{n^{\prime }}+n_{n,k},\;\;\mathrm{if}\;\;1\leq k\leq K,k\neq K. \end{array}$$

Following the principle of NOMA, the power allocation coefficient, $\alpha_{n,k}$, of each UE is expressed as follows:

(7) $$0\leq \alpha_{n,k}\leq 1,\;\sum_{k=1}^{K}\alpha_{n,k}=1,\;\alpha_{n,k}\in \Omega ,$$

where Ω denotes the space of the feasible power allocation coefficient.

### 2.2. Problem Formulation

Based on Equation (5), the signal-to-interference-plus-noise ratio (SINR) for UE${}_{n,k}$ is given by

(8) $$\gamma_{n,k}=\frac{\alpha_{n,k}P_{n}{\left|h_{n,k}w_{n}\right|}^{2}}{I_{n,k}^{U}+I_{n,k}^{N}+\sigma_{n}^{2}},$$

where $I_{n,k}^{U}$ and $I_{n,k}^{N}$ are respectively the intra-beam and inter-beam interference, as follows:

(9) $$\begin{array}{c} I_{n,k}^{U}={\left|h_{n,k}w_{n}\right|}^{2}\sum_{k^{\prime }=k+1}^{K}P_{n}\alpha_{n,k^{\prime }}, \end{array}$$

(10) $$\begin{array}{c} I_{n,k}^{N}=\sum_{n^{\prime }=1,n^{\prime }\neq n}^{N}{\left|h_{n,k}w_{n^{\prime }}\right|}^{2}P_{n^{\prime }}. \end{array}$$

The objective is to maximize the sum rate from all UEs. Thus, the user pairing of each beam $\Phi_{n}$, power allocation coefficient $\alpha_{n,k}$ for each UE, and precoding vector $w_{n}$ should be determined [8]. The problem can then be formulated as follows:

(11) $$\begin{array}{ccc} \max_{\Phi_{n},w_{n},\alpha_{n,k}} & & R^{all}\\ s.t. & \left(C1\right)\;\; & \sum_{k=1}^{K}\alpha_{n,k}=1,\;\alpha_{n,k}\in R,\;n=1,2,\cdots ,N,\\ & \left(C2\right)\;\; & R_{n,k}\geq R_{0},\\ & \left(C3\right)\;\; & |h_{n,k}w_{n}|=0,\;\forall n^{\prime }\neq n, \end{array}$$

where Equation (11) represents the sum rate of the MIMO-NOMA UEs. The constraint of (C1) is the summation of the power allocation coefficients in a beam. The constraint of (C2) means that the BS satisfies the minimum data rate of each UE, $R_{0}$. The constraint of (C3) represents the beamforming constraint. The optimization problem is the non-convex NP-hard. To solve this problem, the computational complexity should be reduced. The precoding matrices can be expressed as follows [5]:

(12) $$W=I_{N},$$

where $I_{N}$ is the N×N identity matrix. Equation (12) represents the inter-beam interference $I_{n,k}^{N}$ can be canceled. Therefore, complex MIMO–NOMA systems can be simplified as single-input single-output (SISO) NOMA systems.

From Equations (8) and  (12), the data rate of UE${}_{n,k}$ can be express as follows:

(13) $$R_{n,k}=\log_{2}\left(1+\frac{\alpha_{n,k}P_{n}{\left|h_{n,k}w_{n}\right|}^{2}}{I_{n,k}^{U}+\sigma_{n}^{2}}\right).$$

UE${}_{n,K}$ is the closest user from the BS, and SIC can be used to remove the intra-beam interference $I_{n,k}^{U}$. Consequently, Equation (13) can be rewritten as follows:

(14) $$R_{n,k}=\left\{\begin{array}{c} \log_{2}\left(1+\frac{\alpha_{n,k}P_{n}{\left|h_{n,k}w_{n}\right|}^{2}}{\sigma_{n}^{2}}\right),\;\;\mathrm{if}\;\;k=K,\;\\ \log_{2}\left(1+\frac{\alpha_{n,k}P_{n}{\left|h_{n,k}w_{n}\right|}^{2}}{I_{n,k}^{U}+\sigma_{n}^{2}}\right),\;\;\mathrm{if}\;\;1\leq k\leq K,k\neq K. \end{array}\right.$$

From Equation (14), the data rate of UEs with 1≤k≤K in a beam can be calculated; the sum rate of all MIMO–NOMA systems can be calculated by summing the data rates of all beams. The sum rate of MIMO–NOMA systems $R^{all}$ can be expressed as follows:

(15) $$R^{all}=\sum_{n=1}^{N}\sum_{k=1}^{K}\log_{2}\left(1+\frac{\alpha_{n,k}P_{n}{\left|h_{n,k}w_{n}\right|}^{2}}{I_{n,k}^{U}+\sigma_{n}^{2}}\right).$$

In the conventional user pairing and power allocation procedure, after the BS acquires the CSI from the UE, the BS determines a pair according to the location or channel gain. This information is transmitted to the UEs. When the response from the UEs has been received, the power allocation coefficient of the UEs belonging to each beam is determined again, and the power is transmitted to each UE.

## 3. Proposed RL-Based Joint User Pairing and Power Allocation

In this section, joint user pairing and power allocation for maximizing the sum rates of a MIMO–NOMA system are proposed. In the wireless channel environment, user pairing and power allocation can be modeled as the repeated interactions between the BS and UEs. The optimal user pairing and power allocation depends on the location of UEs and their radio channel states [18]. The user pairing and power allocation of the BS affect the sum rate of the MIMO–NOMA system. Because the MIMO–NOMA transmission process can be formulated as a Markov decision process, Q-learning can be applied to a MIMO–NOMA system.

**Figure 2:**
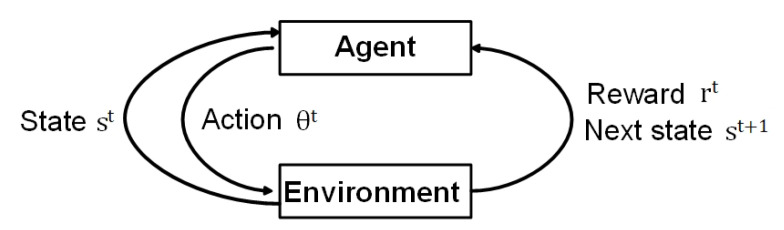
Typical reinforcement learning (RL) architecture.

Q-learning is based on the state, action, and reward [26]. Figure 2 shows a basic structure of RL. In the proposed Q-learning model, the agent is the BS, and the environments is fading, shadowing, and distance environments between the BS and UEs.

### 3.1. Design State and Action

The BS performs the user pairing and power allocation based on Q-leaning, and the Q-function determines the user pairing and power allocation value. The state $s^{t}$ is the quantized channel vector of the UEs ${\hat{h}}_{n,k}$, the action $\theta^{t}$ comprises a user pairing set $\Phi_{n}$ and power allocation coefficient $\alpha_{n,k}$, and the reward is defined as the quantized sum rate ${\hat{R}}^{all}$ of the MIMO–NOMA system. The quantization is performed in *L* steps, and the channel vector of the UEs generated with the Rayleigh distribution is quantized into *L* steps.

The state at time *t* is as follows:

(16) $$s^{t}={\left[{\hat{h}}_{n,k}^{t-1}\right]}_{1\leq n\leq N,1\leq k\leq K}\in \xi ,$$

where ξ is the space of all the possible channel vectors. Moreover, the size of the state space can be expressed as $L^{NK}$.

The action set of the BS is defined as the index of the joint user pairing and power allocation procedure. As assumed in the system model, when there are *M* UEs in the cell and the BS forms *N* beamforming vectors, *K* UEs form a pair in each beam. The user pairing set is defined as $\Phi_{n}$:

(17) $$\Phi_{n}=\left\{\left(n,1\right),\left(n,2\right),\cdots ,\left(n,K\right)\right\},K\geq 2,1\leq n\leq N.$$

When we use the ES method for user pairing, the computational complexity exponentially increases. Meanwhile, if the channel gain of the UEs grouped in the same *n*th pair is assumed to be ordered by Equation (1), the user pairing complexity can be reduced.

Moreover, the power allocation coefficients are quantized into the number of *K* UEs in each beam, and the sum of the power allocation coefficients is set to 1. Thus, Equation (7) can be rewritten as follows:

(18) $$\alpha_{n,k}\in {\left\{k/K\right\}}_{1\leq k\leq K},\sum_{k=1}^{K}\alpha_{n,k}=1,\;\alpha_{n,k}\in \Omega .$$

By multiplying the user pairing index and *K* steps of the power allocation coefficients can be the Q-learning’s joint action. Hence, joint user pairing and power allocation can be performed in one step. From Equations (17) and  (18), the equation of action at time *t* can be expressed as follows:

(19) $$\theta^{t}=\Phi_{n}\times \Omega$$

The size of action spaces is as follows:

(20) $$n\left(\theta^{t}\right)=\left(\begin{array}{c} M\\ N \end{array}\right)K=\frac{M!K}{N!(M-N)!}.$$

From Equation (20), the action set $\theta^{t}$ can be converted into an index set, i.e., $\theta^{t}=\left\{0,1,\cdots ,\left(n\left(\theta^{t}\right)-1\right)\right\}$.

The choice of an action in RL is determined by the tradeoff between exploitation and exploration. In this paper, the action was chosen by applying ϵ-greedy policy and deciding whether to explore with a random action or exploit the action with the best value with the current information according to ϵ. The ϵ-greedy equation is as follows:

(21) $$\theta^{t}=\left\{\begin{array}{cc} \mathrm{argmax}(Q\left(s^{t},\theta^{t}\right)), & \;\;\;\;\;\;\;\mathrm{with}\;\mathrm{probability}\;1-\epsilon \\ \mathrm{random}\;\mathrm{action}, & \;\;\;\;\;\;\;\mathrm{with}\;\mathrm{probability}\;\epsilon . \end{array}\right.$$

**Figure 3:**
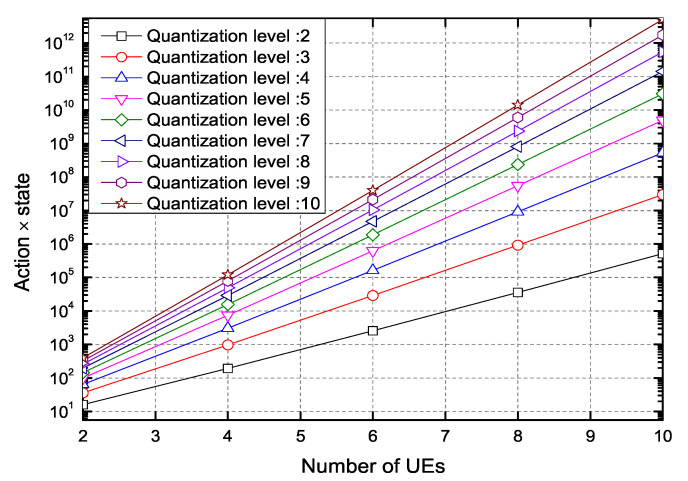
The (action×state) space versus number of user equipment (UEs).

An important point when designing the Q-learning model is the size of the (action×state) space. As the (action×state) space increases, the RL complexity exponentially increases. The number of the quantization level *L* of ${\hat{h}}_{n,k}$ increases the state space. The number of user pairing set due to the number of UEs and the number of quantization levels of the power allocation coefficient affect the action space. The (action×state) space exponentially increases with the number of UEs, as shown in Figure 3. As the quantization level increases, ${\hat{h}}_{n,k}$ approaches to the actual $h_{n,k}$; however, the increase of the quantization levels may be inefficient because the complexity exponentially increases.

**Figure 4:**
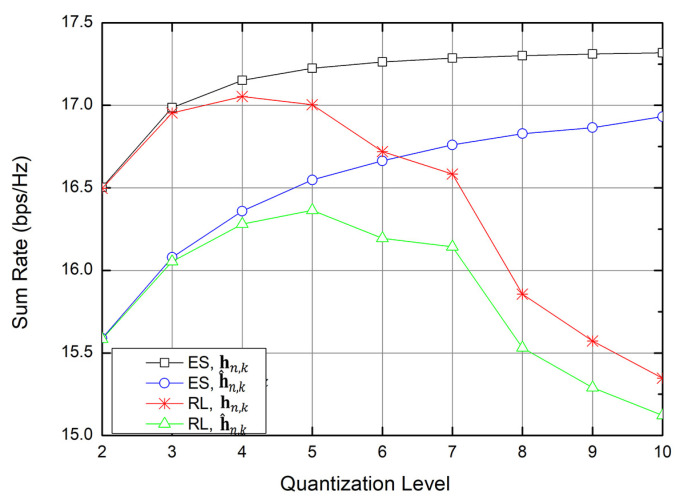
Sum rate versus the number of channel state information (CSI) quantization levels when the time slot is 100,000.

Because of the tradeoff between the complexity and the sum rate, it is important to find the optimal quantization level in the RL structure. Figure 4 shows the sum rate for an increasing quantization level when the time slot is limited to 100,000. The results show that, when the ES scheme is applied, the sum rate increases and converges to about 17.3 bps/Hz. By contrast, when the proposed Q-learning scheme is applied, the sum rate increases and then decreases after a certain level because of the limited time slot (100,000). If the time slot is not limited, the sum rate of Q-learning increases as the quantization level increases. However, as the number of quantization levels increases, the number of states increases, and the RL model requires more time for the sum rate to converge. Our object is to achieve the sum rate similar to that obtained with the ES scheme, while reducing the computational complexity.

In Figure 4, for the case that the reward of RL is calculated with ${\hat{h}}_{n,k}$, the sum rate is highest when the quantization level is 5. Here, we assumed there are four UEs in the cell. For the case that the reward of RL is calculated with $h_{n,k}$, the sum rate is highest when the quantization level is 4. Here, ${\hat{R}}^{all}$, which the reward of RL, is calculated with ${\hat{h}}_{n,k}$, and $R^{all}$, which is the sum rate, is calculated with $h_{n,k}$. The difference between ${\hat{R}}^{all}$ and $R^{all}$ is due to the quantization error in the CSI. Because the object is to increases the sum rate, we chose the quantization level as 4 in the proposed Q-learning.

### 3.2. Q-Learning-Based Joint User Pairing and Power Allocation Procedure

The reward is the sum rate of the MIMO–NOMA UEs. From Equation (15) reward at time *t* can be expressed as follows:

(22) $${\hat{R}}^{all}=\sum_{n=1}^{N}\sum_{k=1}^{K}\log_{2}\left(1+\frac{\alpha_{n,k}P_{n}{\left|{\hat{h}}_{n,k}w_{n}\right|}^{2}}{I_{n,k}^{U}+\sigma_{n}^{2}}\right),$$

where ${\hat{R}}^{all}$ is the sum rate calculated with ${\hat{h}}_{n,k}$. In Q-learning, ${\hat{R}}^{all}$ is continuously updated by Q-function; whereas $R^{all}$ is calculated with $h_{n,k}$. The user pairing index and power allocation coefficient is simultaneously determined by using Q-learning.

Moreover, Q(s,θ) denotes the Q-function of the BS for system state *s* and action θ:

(23) $$Q\left(s^{t},\theta^{t}\right)\leftarrow \left(1-\beta \right)Q\left(s^{t},\theta^{t}\right)+\beta \left[r\left(s^{t},\theta^{t}\right)+\delta \max_{\theta^{\prime }}Q\left(s^{t+1},\theta^{t}\right)\right],$$

where the learning rate β∈(0,1] represents the weight of the recent experience in the learning process. The discount factor δ∈[0,1] controls the importance of the immediate and future rewards.

**Figure 5:**
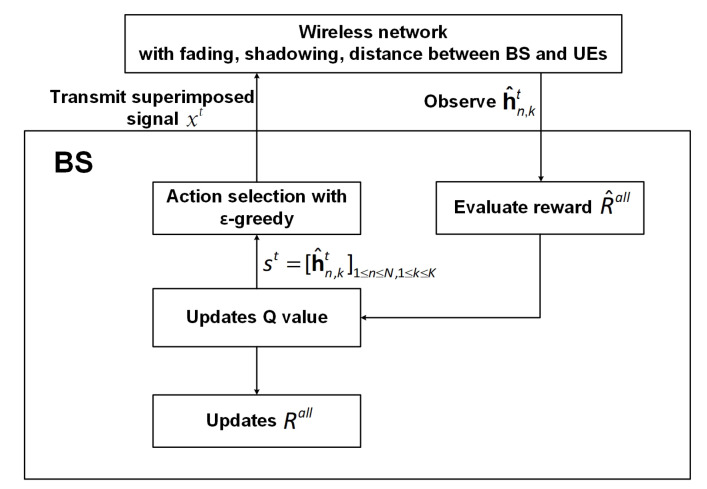
Illustration of the Q-learning-based joint user pairing and power allocation scheme.

The main structure of the joint user pairing and power allocation based on Q-learning is illustrated in Figure 5 and the algorithm is summarized in Algorithm  1.
**Algorithm 1** Joint user pairing and power allocation with Q-learning1:Set $Q\left(s^{t},\theta^{t}\right)=0,\forall \theta^{t}=0$ and $\forall s^{t}=0$   2:**for**t=1 to *T* **do**   3:    Observe the current state $s^{t}$   4:    Choose action $\theta^{t}$ in Equation (19)   5:    Convert action into user pairing set $\Phi_{n}$ and power allocation coefficient $\alpha_{n,k}^{t}$   6:    **for** n=1 to *N* **do**   7:        **for** k=1 to *K* **do**   8:           Allocate the transmit power $\alpha_{n,k}^{t}P_{n}$ and pair $\Phi_{n}$ for the signal to user *k*   9:        **end for**  10:    **end for**  11:    Send the superimposed signal $x^{t}$ via *N* antennas   12:    Observe fading, shadowing, and the distance between BS and UEs   13:    Observe the CSI $h_{n,k}^{t}$   14:    Calculate the reward ${\hat{R}}^{all}$   15:    $s^{t+1}={\left[{\hat{h}}_{n,k}^{t}\right]}_{1\leq n\leq N}$   16:    Update $Q(s^{t},\theta^{t})$ in Equation (23)   17:    Calculate $R^{all}$ in Equation (15)   18:**end for**  

Algorithm  1 works as follows: First, the Q-learning parameters, $Q(s^{t},\theta^{t})$, $\theta^{t}$, and $s^{t}$, are initialized. In Step 3, the BS observes the current state $s^{t}$. In Step 4, the BS selects the action $\theta^{t}$ according to the ϵ-greedy policy. In Step 5, the BS converts the selected $\theta^{t}$ into a user pairing set $\Phi_{n}$ and the power allocation coefficient $\alpha_{n,k}$. In Step 10, the BS transmits the superimposed signal $x^{t}$ via *N* antennas to the UEs. In Step 12, the BS observes fading, shadowing, and the distance between BS and UEs. In Step 13, the CSI $h_{n,k}^{t}$ is observed, and in Step 14, the reward ${\hat{R}}^{all}$ is calculated. In Step 15, the next state $s^{t+1}$ is quantized. Finally, in Steps 16 and 17, the BS updates $Q(s^{t},\theta^{t})$ and $R^{all}$ based on Equations (23) and  (15), respectively.

## 4. Numerical Results

We consider a MIMO-NOMA system with one BS. The BS is located at the center. The UEs are randomly distributed in a cell within a radius of 50 to 500 m. To take the movement and the channel fluctuation of each UE into consideration, the location and the CSI of each UE is randomly generated in every time slot. In addition, two UEs are assumed to be paired in one beam; Equation (15) can then be expressed as follows:

(24) $$\begin{array}{c} R^{all}=\sum_{n=1}^{N}\left(\log_{2}\left(1+\frac{\alpha_{n,1}P_{n}{\left|h_{n,1}w_{n}\right|}^{2}}{I_{n,1}^{U}+\sigma_{n}^{2}}\right)\log_{2}\left(1+\frac{\alpha_{n,2}P_{n}{\left|h_{n,2}w_{n}\right|}^{2}}{\sigma_{n}^{2}}\right)\right). \end{array}$$

Because K=2, the power allocation coefficient can be quantized into level 2. The power allocation coefficient set Ω is assumed to be Ω=[0.2,0.4]. The learning rate of the Q-function is set to 0.9999, and the discount factor is set to 0.0001. The time slot is one TTI, e.g., 1 ms, in a LTE system or a 5G system with 15 kHz subcarrier spacing [27]. At every time slot, the BS observes the CSI of UEs and performs the user pairing and power allocation. The total number of time slots is 100,000; the simulation results are obtained by repeating 1000 times under iteration. The simulation parameters used in this paper are listed in Table 2.

The simulation was performed with the following simulation environments: Intel(R) Core i9−9900K CPU @3.60 GHz, RAM 16.0 GB, Window10, python 3.7, GPU GeForce RTX 2080 Ti.

The performance of the proposed RL based scheme is compared with the following schemes: the ES, OMA, random selection, and phased RL schemes for determine the user pairing and the transmit power of UEs. In the ES scheme, the user paring and the transmit power are optimally determined by using the exhaust search method, and therefore the ES scheme shows the highest performance. In the random selection scheme, the BS randomly determines the user pairing and the transmit power of UEs. In the OMA scheme, the BS serves only one UE in a beam and therefore the sum rate is given by [28]

(25) $$\begin{array}{c} R_{OMA}=\sum_{n=1}^{N}\sum_{k=1}^{K}\left(\frac{1}{k}\log_{2}\left(1+\frac{P_{n}{\left|h_{n,k}w_{n}\right|}^{2}}{\sigma_{n}^{2}}\right)\right). \end{array}$$

In the phased RL-based user paring and power allocation scheme, the BS sequentially determines a user pairing and the transmit power of UEs. That is, after pairing the UEs, the BS can then determine the transmit power of UEs. In the phased RL scheme, the Q-function for user pairing is defined as $Q_{UP}\left(s,\theta_{UP}\right)$ and the Q-function of the power allocation is defined as $Q_{PA}\left(s,\theta_{PA}\right)$. From Equation (17), action of user pairing RL is defined as $\theta_{UP}=\Phi_{n}$. From Equation (18), action of power allocation RL is defined as $\theta_{PA}=\alpha_{n,k}$. First, user pairing RL proceeds in which the rewards are only used to update the Q-function, where the reward is calculated with the fixed power allocation. The user pairing set $\Phi_{n}$ is determined by the BS through $Q_{UP}\left(s,\theta_{UP}\right)$. In power allocation RL, the user pairing set $\Phi_{n}$ is observed as a state along with ${\hat{h}}_{n,k}$. Power allocation coefficient is determined by the BS through $Q_{PA}\left(s,\theta_{PA}\right)$. Finally, the BS updates $Q_{PA}\left(s,\theta_{PA}\right)$, and $R^{all}$. The algorithm of the phased RL-based user pairing and power allocation scheme is summarized in Algorithm  2.
**Algorithm 2** Phased RL-based user pairing and power allocation1:Set $Q_{UP}\left(s_{UP}^{t},\theta_{UP}^{t}\right)=0,\forall \theta_{UP}^{t}=0$ and $\forall s_{UP}^{t}=0$   2:Set $Q_{PA}\left(s_{PA}^{t},\theta_{PA}^{t}\right)=0,\forall \theta_{PA}^{t}=0$ and $\forall s_{PA}^{t}=0$   3:**for**t=1 to *T* **do**   4:    Choose action $\theta_{UP}^{t}$ in Equation (17)   5:    **for** n=1 to *N* **do**   6:        **for** k=1 to *K* **do**   7:           Allocate the fixed transmit power for the signal to user *k*   8:        **end for**  9:    **end for**  10:    Send the superimposed signal $x^{t}$ via *N* antennas   11:    Observe $s^{t}$ and reward ${\hat{R}}_{UP}^{t}$   12:    Update $Q_{UP}\left(s_{UP}^{t},\theta_{UP}^{t}\right)$ in Equation (23)   13:    Choose action $\theta_{PA}^{t}$ in Equation (18)   14:    **for** n=1 to *N* **do**   15:        **for** k=1 to *K* **do**   16:           Apply user pairing $\theta_{UP}^{t}$   17:           Allocate the transmit Power $\alpha_{n,k}^{t}P_{n}$ for the signal to user *k*   18:        **end for**  19:    **end for**  20:    Observe reward ${\hat{R}}_{PA}^{t}$   21:    $s^{t+1}={\left[{\hat{h}}_{n,k}^{t}\right]}_{1\leq n\leq N}$   22:    Update $Q_{PA}\left(s_{PA}^{t},\theta_{PA}^{t}\right)$ in Equation (23)   23:    Calculate $R^{all}$ in Equation (15)   24:**end for**  

**Figure 6:**
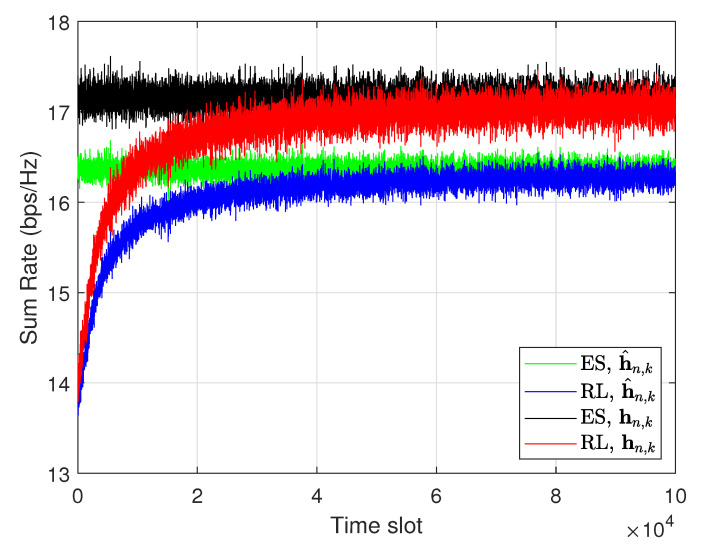
Sum rate of the RL scheme.

Figure 6 shows the sum rate of the RL scheme with respect to the time slot, when the number of UEs is 4 and the quantization levels of CSI is 4. The transmit power of the BS is 43 dBm. In the RL-based scheme, the actions are randomly determined in the first time, which leads to a lower sum rate. As time elapses, the sum rate of the RL-based scheme increases and when the time slot reaches about 40,000, it approximately converges to that of the ES scheme with a performance difference of 0.57%. It also means that it takes about 40 seconds (when the time slot is 1 ms) to achieve the sum rate similar to ES. However, the proposed RL-based scheme can keep up with the changing radio channel of the UE because the BS continuously trains the machine for every time slot. Hence, if the wireless channel environment of the UE does not change very rapidly, the proposed RL-based scheme can be applied to real-time scenarios. Because of the quantization error, the RL’s reward is lower than the sum rate calculated with the $h_{n,k}$. The numerical results are compared with those of other schemes by the sum rate calculated with $h_{n,k}$.

**Figure 7:**
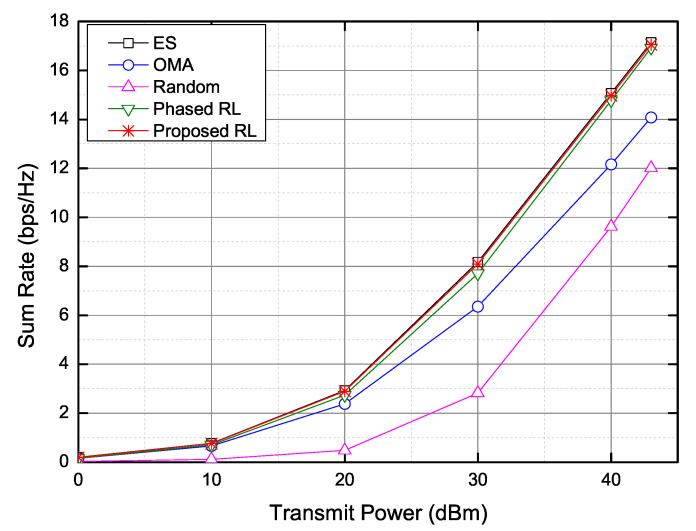
Sum rate versus transmit power.

When the transmit power of the BS increases, the sum rate increases, as shown in Figure 7. As the transmit power of the BS increases, the sum rates of all schemes increase. The random selection scheme shows the worst sum rate because the SIC is not perfect. As presented in Figure 7, the proposed scheme shows approximately same results as the ES, and also the phased RL scheme exhibits a similar sum rate. When the transmit power is 43 dBm, the proposed RL scheme increases the sum rate by about 21.15% and about 41.98% in comparison with the OMA scheme and the random selection scheme, respectively.

**Figure 8:**
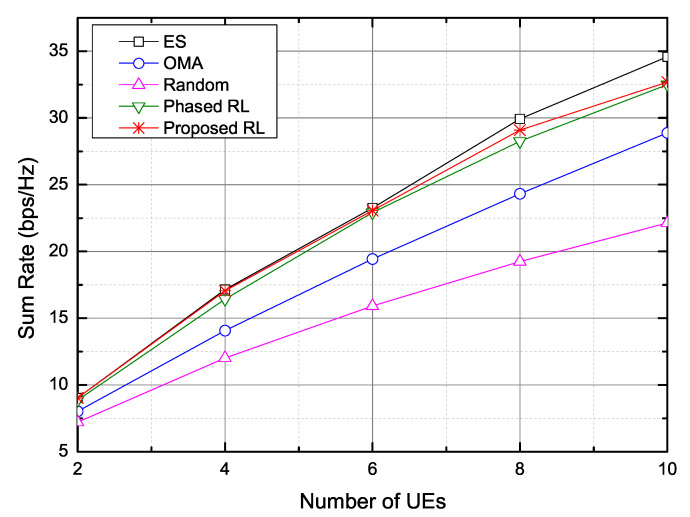
Sum rate versus the number of UEs.

Figure 8 shows the sum rate as the number of UEs increases. As the number of UEs increases, the sum rates of all schemes increase and finally gradually converge. The performance difference between the ES scheme and the proposed scheme slightly increases as the number of UEs increases. For 10 UEs, the performance difference is about 5.48%, which is due to the increased size of states. The proposed scheme increases the sum rate by about 13.17% and about 47.67% in comparison with the OMA scheme and the random selection scheme, respectively. However, the proposed scheme and the phased RL scheme show the similar performance.

**Figure 9:**
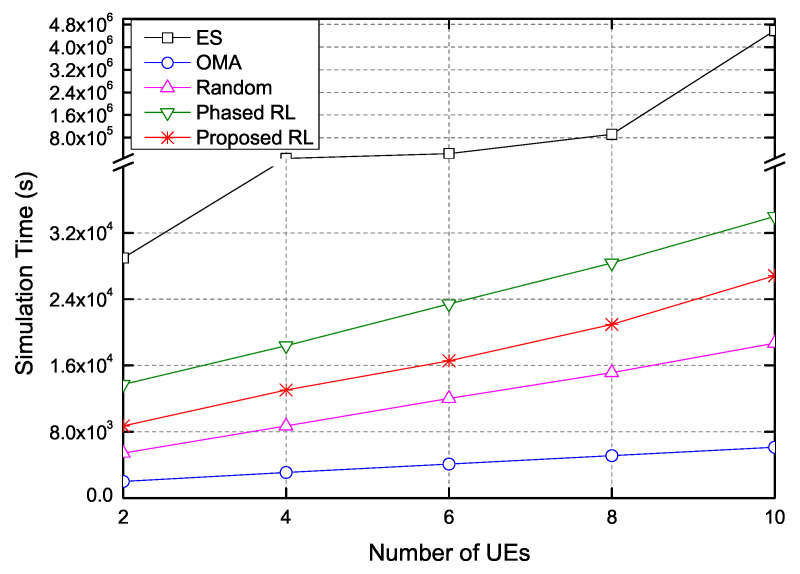
The total simulation time for 1000 iterations versus the number of UEs.

Figure 9 presents the required simulation time as the number of UEs increases. Because the ES scheme investigates all possible actions, its simulation time is extremely high. The results show that the proposed scheme is more efficient than the phased RL scheme in terms of the time complexity. The proposed scheme reduces the time complexity by about 20.97% compared with the phased RL scheme.

The proposed scheme reduces the computational complexity. The ES scheme finds all possible actions and therefore, when the action space is denoted by $n=\theta^{t}$, the complexity of the ES scheme is represented by O(n). The phased RL scheme sequentially determines the user paring and the transmit power of UEs in each pair. Hence, the complexity of the phased RL can be expressed as 2·O(1), because the RL requires a complexity of O(1) after it converges. The proposed RL-based scheme calculates the reward by choosing one action and therefore it has a complexity of O(1).

## 5. Conclusions

In this paper, an RL-based joint user pairing and power allocation scheme for MIMO–NOMA systems is proposed. To reduce the computational complexity of finding the user pairing and the transmit power of users, the Q-learning was applied. The user pairing and the transmit power allocation were simultaneously performed in Q-learning’s action. The proposed scheme shows the sum rate similar to that of the ES scheme with the low computational complexity. The proposed scheme reduces the time complexity compared with the phased RL scheme although they show the similar performance in terms of the sum rate. However, as the number of UEs increases, the performance difference between the proposed scheme and the ES scheme slightly increases. In the future, we will apply the DQN to the MIMO-NOMA system in order to reduce the performance difference.

## Figures and Tables

**Table 1:** Symbols and description.

Symbol	Description
*M*	Total number of users
*n*	Number of BS antennas
*k*	Number of users in a beam
$P_{B}$	Total power of the BS
$P_{n}$	Transmit power at the *n*th beam
$s_{n,k}$	Signal transmitted to the *k*th UE at the *n*th beam
$x_{n}$	Superimposed signal at the *n*th beam
$h_{n,k}$	Channel vector to the *k*th UE at the *n*th beam
${\hat{h}}_{n,k}$	Quantized channel vector to the *k*th UE at the *n*th beam
$d_{n,k}$	Distance between BS and the *k*th UE at the *n*th beam
$w_{n}$	Precoding vector at the *n*th beam
$\gamma_{n,k}$	SINR of the *k*th UE at the *n*th beam
$R_{n,k}$	Data rate of the *k*th UE at the *n*th beam
$R^{all}$	Sum rate of MIMO-NOMA systems
${\hat{R}}^{all}$	Sum rate of MIMO-NOMA systems using quantized channel vector
$\Phi_{n}$	The user pairing set at the *n*th beam
$\alpha_{n,k}$	Power allocation coefficient to the *k*th UE at the *n*th beam
η	Path loss exponent
$n_{n,k}$	Addictive white gaussian noise (AWGN) to the *k*th UE at the *n*th beam
*L*	Number of CSI quantization level
$I_{n,k}^{N}$	Inter-beam interference to the *k*th UE at the *n*th beam
$I_{n,k}^{U}$	Intra-beam interference to the *k*th UE at the *n*th beam
*s*	State of Q-learning
θ	Action of Q-learning
*r*	Reward of Q-learning
β	Learning rate
δ	Discount factor

**Table 2:** Simulation parameters.

Parameter	Value
Total number of UEs, *M*	2, 4, 6, 8, 10
Number of transmit antennas, *N*	1, 2, 3, 4, 5
Number of UEs in a beam, *K*	2
Power allocation coefficient, $\alpha_{n,k}$	0.2, 0.4
Path loss coefficient, η	3
Learning rate, β	0.9999
Discount factor, δ	0.0001
Time slot (1 ms), *T*	100,000
Number of iterations, *I*	1000
